# On Stammering

**Published:** 1841-06-26

**Authors:** J. Trudeau

**Affiliations:** New York


*On Stammering*. By J. Trudeau, M. D., of New York.

The section of the genio glossial muscles for the cure of stammering was performed a few days ago, in this city, by Dr. Alfred C. Post, one of our most distinguished and enterprising surgeons. The operation, exceedingly simple, was executed in the following manner:—The patient being seated on a low stool, the head supported by an assistant, the mouth was widely opened, and the tongue drawn by the left hand of the same assistant, upwards and slightly forwards, so as to put the muscles upon the stretch. The surgeon, seizing then a pair of sharp pointed and strong scissors, plunged the instrument close to the bone, with the blades opened so as to divide four lines on each side of the frænum. The point of the scissors being distinctly felt above the insertion of the genio hyoid muscle, the blades were rapidly closed, dividing by a single stroke the mucous membrane, the insertion of the frænum, and the tendon of the genio glossi. The finger introduced in the wound satisfied the assistant that the tendon had been entirely divided. Blood flew freely, but was arrested by the application of cold and astringents.

Having not seen the patient since, I am not able to speak of the results. As Dr. Post will shortly publish the case, I will refer to the New York Journal of Medicine and Surgery. It is, I think, the first operation of the kind performed in the United States.

